# Remediation Strategies for Mycotoxins in Animal Feed

**DOI:** 10.3390/toxins15090513

**Published:** 2023-08-22

**Authors:** Jiang Deng, Jun-Cheng Huang, Ze-Jing Xu, Ying Liu, Niel Alexander Karrow, Meng Liu, Lv-Hui Sun

**Affiliations:** 1 State Key Laboratory of Agricultural Microbiology, Hubei Hongshan Laboratory, Frontiers Science Center for Animal Breeding and Sustainable Production, College of Animal Sciences and Technology, Huazhong Agricultural University, Wuhan 430070, China; jiangdeng@webmail.hzau.edu.cn (J.D.);; 2 Hubei Jin Xu Agricultural Development Limited by Share Ltd., Wuhan 430015, China; 3 Tianjin Animal Disease Prevention and Control Center, Tianjin 300402, China; 4 Department of Animal Biosciences, University of Guelph, Guelph, ON N1G2W1, Canada

Mycotoxins occur widely in various animal feedstuffs, with more than 500 mycotoxins identified so far [1]. Generally, aflatoxin B1 (AFB1), zearalenone (ZEN), deoxynivalenol (DON), fumonisin B1 (FB1), ochratoxin A (OTA) and T-2 toxin are the most common mycotoxins that occur in animal feed, such as corn, wheat, barley, peanuts, oats, rice and their by-products. These mycotoxins have cytotoxicity, hepatoxicity, genotoxicity, immunotoxicity, reproductive toxicity and gastrointestinal toxicity [2,3,4,5,6,7]; as a result, ingestion of contaminated feedstuffs can seriously threaten the animal’s health, production, and the quality and safety of their products [8,9,10]. Wei et al. found that contamination with aflatoxins, trichothecenes type B, fumonisins and ZEN was present in more than 13, 88, 80 and 79% of raw material and feeds, respectively, during 2021 in China [11]. Additionally, Eskola et al. analyzed more than 500,000 studies of mycotoxin contamination and found that the occurrence of mycotoxins above the detectable level was up to 60~80% [12].

Therefore, the development of counteracting strategies for mycotoxin control has received increasing attention from scientists and the feed industry [1,5]. This Special Issue has collected research articles and reviews focused on recent advances in decontamination of these common mycotoxins in feed. In particular, this issue contains papers related to (1) novel materials or novel microorganisms which can biodegrade the mycotoxins; (2) novel modified adsorbents to reduce the toxicity of mycotoxins in livestock and poultry; (3) nutritional strategies to help mitigation of mycotoxicosis; and (4) better understanding the toxicity mechanisms of mycotoxins to provide a theoretical basis for the development of antidotes (Figure 1).

Novel materials or novel microorganisms: At present, sorption and degradation are applied to reduce the mycotoxin content in animal feeds. In particular, He et al. overcame the lower efficiency of light utilization and photocatalytic degradation ability of titanium dioxide (TiO_2_) with cerium (Ce) doping and successfully synthesized a photocatalytic nanomaterial, Ce-doped TiO_2_, using the sol–gel method. These novel nanomaterials, especially 0.5Ce-TiO_2_, showed the most effective photocatalytic degradation of DON under UV light (λ = 254 nm) in aqueous solution [13]. Additionally, silver nanoparticles showed a strong antifungal effect on *Aspergillus* species, while iron nanoparticles presented a greater adsorption ability with AFB1 [14]. Similarly, carbon nanotubes were generally decorated with various nanoparticles, such as silver and zinc oxide, to suppress the fungal activity, thus reducing the generation of mycotoxins indirectly [15].

Biological methods include metabolization by microorganisms, degradation by secreted enzymes and microbial adsorption. Xu et al. summarized that some bacteria (*Lactobacills*, *Bacillus*, *Sphingomonas*) and fungi (*Aspergillus*, yeast *Saccharomyces* and *Propionibacterium*) could degrade or remove mycotoxins in food and feed. Furthermore, catalase, lactose hydrolase, 3-O-acetyltransferase and fumonisin carboxylesterase FumD could also degrade AFB1, ZEN, DON and FB1, respectively [16,17,18].

Novel modified adsorbents: It is well known that AFB1 can be partly bio-transformed into AFM1 in dairy cows, which is then secreted into the milk, thus leading to serious concerns regarding milk product safety and human health. Therefore, Cha et al. investigated the AFM1 residue in milk when supplemented with a moderate concentration of AFB1 (8 µg/kg), and found that the addition of the adsorbent, which consists of montmorillonite, diatomite, yeast cell wall extracts and sodium alginate, reduced the AFM1 residue content in milk [19]. However, children aged 2–11 years still faced a potential risk to the liver despite a significantly lower estimated daily intake (EDI) and hazard index (HI) [19], which suggests that we should pay more attention to residue hazards and develop more efficient novel adsorbent materials.

Nutritional strategies: To reduce the contamination and adverse effect of mycotoxins, throughout the entire process from the farm to the table, as efficiently as possible, nutrients are generally applied during livestock and poultry production to alleviate the toxicity, such as selenium, vitamins, functional amino acids (DL-selenomethionine, glutamic acid, arginine, etc.), and plant extracts (resveratrol, astaxanthin, curcumin, silymarin and soybean isoflavone, etc.) [10,20,21,22]. DL-selenomethionine, as an organic selenium compound, attenuated ZEN-induced ROS production and lipid peroxidation, and it increased the antioxidant capacity in porcine intestinal epithelial J2 (IPEC-J2) cells through the Nrf2/Keap1-ARE signaling pathway [20]. Similarly, Xu et al. found that resveratrol alleviated ZEN-induced cytotoxicity, oxidative stress and apoptosis via the PI3K/Akt-mediated Nrf2/HO-1 signaling pathway in the TM4 cell model [21]. Astaxanthin (AST) was found to attenuate OTA-induced cytotoxicity, oxidative damage and apoptosis in mouse kidney by activating the Nrf2/KEAP1 signaling pathway [22]. Selenium, which plays important roles in immunity, antioxidant defense and detoxification, could mitigate the AFB1-induced cardiotoxicity through four selenoproteins and ferroptosis activator (SLCA2 and SLCA11) signaling in chicks [23,24]. In conclusion, most of the above nutrients can mitigate the adverse effects of mycotoxins in animals, mainly through anti-inflammation, antioxidant defense and anti-apoptosis [21,25,26,27,28].

Better understanding of mycotoxins: Several new toxic mechanisms of mycotoxins and detoxification have been discovered. Glutathione S-transferases, belonging to the phase II metabolizing enzymes, play important roles in the detoxification of drugs and mycotoxin in animal liver. Zhang et al. conducted a study on alpha-class GST involved in the detoxification of AFB1 in duckling liver and found that overexpression of GST and GST3 could increase the formation of AFBO-GSH by 47.0 or 13.4%, respectively, compared to the non-detoxification group [29]. Zhang et al. identified that BACH1 was a crucial gene for AFB1 toxicity through genome-wide CRISPR/Cas9 knock-out screening in porcine kidney cells, and discovered that the small molecules 1-piperazineethanol and α-[(1,3-benzodioxol-5-yloxy)methyl]-4-(2-methoxyphenyl) (M2) could alleviate weight loss and oxidative and liver injury induced by AFB1 in mice [30]. Specifically, Yuan et al. found that low-dose dietary ZEN (750 μg/kg) could reduce production performance, ovarian function and intestinal microbes in laying hens via dysregulation of gut microbes; and their study indicated that *g_norank_f_Barnesiellaceae*, *g_Hydrogenoanaerobacterium* and *g_Butyricmonas* showed a positive correlation with production performance, egg quality and (or) ovarian function [31]. Notably, the novel toxicity of trichothecene mycotoxin has been reported by Wenda Wu’s group [32,33,34,35,36]. Specifically, T-2 toxin-induced emetic response showed a correlation with the secretion of the intestinal hormones glucagon-like peptide_-17–36_ (GLP-1) and glucose-dependent insulinotropic polypeptide (GIP) mediated by calcium transduction. Furthermore, suppression of the calcium-sensitive receptor (CaSR) and transient receptor potential (TRP) channels alleviated emesis by their antagonists NPS-2143 and ruthenium red in mink [32]. Similarly, DON-3-glucoside (D3G), which co-occurs with DON, could also trigger marked emesis via the exocytosis of brain–gut peptides GIP and substance (SP); additionally, the GIP and neurokinin 1 receptor (NK-1R) are potential targets to diminish the intestinal emetic response [33]. An anorexic response, caused by trichothecene A (T-2 toxin, HT-2 toxin, diacetoxyscripenol and neosolaniol, and trichothecene B DON) and its congeners (DON, 3-acetyldeoxynivalenol, 15-acetyldeoxynivalenol, fusarenon X and nivalenol), was associated with the generation of GLP-1 and cholecystokinin (CCK) or SP, which could be suppressed by the Exending_9–39_ CCK antagonist (Sigma-Aldrich, St. Louis, MO, USA) and Emend^®^ (Merck& Co, Inc, Kenilworth, NJ, USA) [34,35,36]. To summarize, gastrointestinal vomiting and anorexia caused by trichothecene mycotoxins are usually related to the release of brain–gut peptides GLP-1, GIP and SP, and the remediation measures can take CaSR and TRP channels as well as NK-1R and GLP-1R into consideration [32,33,34,35,36]. Liu et al. revealed that T-2 toxin-induced intestinal damage was associated with an alteration in nucleotide and glyceropholipid metabolism, redox homeostasis and apoptosis, which accounted for intestinal damage in chicken [37]. Overall, these findings provide novel ideas for the development of remediation strategies for the control of mycotoxins in animal feed. T-2 toxin also induces cell damage through DNA damage and repair, as well as oxidative-stress-mediated apoptosis [37]. Interestingly, cell death induced by DON in piglet intestines was also related to ferroptosis, besides apoptosis and pyroptosis, which appears to be a novel mechanism [38,39]. Additionally, Liu et al. reported that DON could induce cell necrosis and hepatoxicity through the upregulation of CYP enzymes mediated by DNA methylation in L02 cells and piglets; another novel pathway [40]. AFB1 induced cell death via apoptosis and ROS generation in Leghorn male hepatoma (LMH), IPEC-J2 and porcine alveolar macrophage (3D4/21) cells [41]; moreover, OTA-, FB1- and ZEN-induced cycotoxicity was also related to apoptosis, pyroptosis or necroptosis in vivo and/or in vitro [42,43,44]. A better understanding of the toxic mechanism of those mycotoxins will provide a potential target to develop detoxification strategies, thereby contributing to the production of livestock and poultry.

In summary, mycotoxins have been a non-negligible problem in the livestock and poultry industry, and have an impact on food safety as well as human health due to their worldwide contamination, causing substantial economic losses [45,46]. It is necessary to develop novel adsorbing materials and nutrients, as well as comprehensively analyze the toxicity mechanisms to prevent mycotoxicosis. In our opinion, prevention and remediation of mycotoxins in the future should focus on two dominant aspects: (1) continue developing novel effective adsorbents or biodegrading enzymes to reduce mycotoxins in food and feed; (2) continue investigating bioactivity nutrients to be applied for detoxification of mycotoxins in vivo. Through these measures, we hope to minimize the ingestion of mycotoxins in feeds and protect animals from being damaged by the use of various nutrients as much as possible.

## Figures and Tables

**Figure 1:**
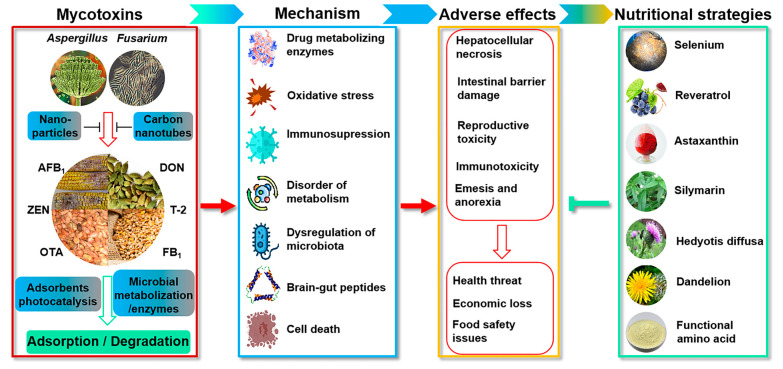
Occurrence, toxicity and control of mycotoxins in animal feeds.

## Data Availability

Not applicable.
